# A nomogram integrating hemodynamic and inflammatory markers to predict early neurological deterioration after tenecteplase in branch atheromatous disease: an early in-hospital risk stratification model

**DOI:** 10.3389/fneur.2026.1806830

**Published:** 2026-07-27

**Authors:** Changhong Yuan, Qingsong Zhang, Yingchun Zhu, Qiaoyun Zhou, Qun Liu, Lu Zhang

**Affiliations:** 1 Department of Neurology, Anhui No. 2 Provincial People’s Hospital, Hefei, Anhui Province, China; 2 Department of Neurology, The People’s Hospital of Bozhou, Bozhou, Anhui Province, China; 3 Department of Respiratory Medicine, Anhui No. 2 Provincial People’s Hospital, Hefei, Anhui Province, China

**Keywords:** blood pressure variability, blood urea nitrogen to albumin ratio, branch atheromatous disease, early neurological deterioration, nomogram, systemic inflammatory response index, tenecteplase, thrombolysis

## Abstract

**Objective:**

To investigate the risk factors for early neurological deterioration (END) in patients with branch atheromatous disease (BAD) receiving intravenous tenecteplase thrombolysis, and to construct a predictive nomogram model.

**Methods:**

Clinical data of BAD patients receiving intravenous tenecteplase thrombolysis from September 2021 to October 2025 were retrospectively collected from Anhui No. 2 Provincial People’s Hospital (training set, *n* = 193) and Bozhou People’s Hospital (external validation set, *n* = 265). END was defined as an increase of ≥1 point in the motor items of the National Institutes of Health Stroke Scale (NIHSS) or an increase of ≥2 points in the total score within 72 h after onset. Least absolute shrinkage and selection operator (LASSO) regression and multivariate logistic regression were used to identify relevant factors and construct the nomogram. Model performance was evaluated using receiver operating characteristic curves, calibration curves, and clinical impact curves. Optimism correction was performed using 500 bootstrap resampling, and model generalizability was validated using an external validation set.

**Results:**

LASSO regression identified four predictors: 24-h systolic blood pressure coefficient of variation (CV-24hSBP), fasting blood glucose (FBG), systemic inflammatory response index (SIRI), and blood urea nitrogen to albumin ratio (BAR). Multivariate logistic regression showed that CV-24hSBP (OR = 2.704, 95% CI: 1.471–4.967, *p* = 0.001), FBG (OR = 1.253, 95% CI: 1.016–1.547, *p* = 0.034), SIRI (OR = 3.140, 95% CI: 1.584–6.223, *p* < 0.001), and BAR (OR = 1.480, 95% CI: 1.207–1.815, *p* < 0.001) were independent factors associated with END. The apparent AUC of the model in the training set was 0.850 (95% CI: 0.796–0.904); after bootstrap optimism correction, the corrected AUC was 0.837 (95% CI: 0.780–0.889). The AUC in the external validation set was 0.824 (95% CI: 0.778–0.879). Calibration curves and clinical impact curves demonstrated good calibration and clinical utility of the model.

**Conclusion:**

The nomogram model based on CV-24hSBP, FBG, SIRI, and BAR demonstrates good predictive performance and generalizability for END in BAD patients receiving intravenous tenecteplase thrombolysis. Because CV-24hSBP requires continuous blood pressure monitoring for 24 h after thrombolysis, this model is not suitable for pre-thrombolysis or ultra-early prediction but can serve as an in-hospital early risk stratification tool to identify high-risk patients and guide individualized intervention.

## 1 Introduction

Since the concept of branch atheromatous disease (BAD) was proposed by Professor Caplan in 1989 (1), the theory has been further enriched and refined through the etiological and pathogenetic classification of acute ischemic stroke in the Trial of Org 10,172 in Acute Stroke Treatment (TOAST), as well as the subsequent supplementation by the Chinese Ischemic Stroke Subclassification (CISS) (2). In clinical practice, with the widespread application of techniques such as CTA and high-resolution magnetic resonance imaging, the diagnosis of BAD has become increasingly easier, and BAD has emerged as a common type of acute ischemic stroke in Asian populations, accounting for approximately 10–15% of all acute ischemic stroke cases (3, 4). Patients with BAD often present with capsular warning syndrome or pontine warning syndrome, and their clinical symptoms tend to progress and worsen (5). In recent years, with the continuous development of stroke center systems nationwide, an increasing number of patients with acute ischemic stroke have been able to receive standardized treatment. The popularization of intravenous thrombolysis for acute ischemic stroke has significantly advanced, and intravenous thrombolysis in patients with low NIHSS scores has gradually gained widespread acceptance, among whom a large proportion are BAD patients. However, clinical practice has revealed that these patients still face a high risk of early neurological deterioration (END) after receiving intravenous thrombolysis. Studies from Asian populations have shown that the incidence of END in BAD patients ranges from 37 to 46.7% (6, 7), and whether intravenous thrombolysis can reduce the occurrence of END remains controversial (8, 9). Early identification of high-risk patients for END and the provision of individualized interventions are of significant clinical importance. This study aims to explore the factors associated with END in BAD patients after intravenous thrombolysis, hoping to provide a scientific basis for clinical practice.

## 2 Materials and methods

### 2.1 Inclusion and exclusion criteria

We retrospectively analyzed the medical records of patients with BAD who received intravenous tenecteplase thrombolysis via the stroke green channel at the Department of Neurology, Anhui No. 2 Provincial People’s Hospital from September 2021 to October 2025. Medical records of patients with BAD who received intravenous tenecteplase thrombolysis at the Bozhou People’s Hospital during the same period were also collected as an external validation set (with the same inclusion and exclusion criteria).

Inclusion criteria were as follows: (1) Diagnosis of BAD conforming to the Chinese expert consensus criteria, specifically: for ischemic stroke in the lenticulostriate artery (LSA) territory, diffusion-weighted imaging (DWI) showed infarct lesions involving three or more levels in the corresponding blood supply area; for ischemic stroke in the paramedian pontine artery (PPA) territory, DWI showed an infarct lesion connected to the ventral pontine surface, located near the midline, confined to one side and not crossing the midline; (2) Age between 18 and 85 years; (3) Receipt of intravenous tenecteplase thrombolysis within 4.5 h of symptom onset.

Exclusion criteria were as follows: (1) Previous history of cerebral infarction with significant residual sequelae and a pre-thrombolysis modified Rankin Scale (mRS) score ≥2; (2) Non-standard dosage of tenecteplase or incomplete clinical data, including failure to collect venous blood for laboratory testing within 24 h of admission; (3) Presence of fever or evidence of infection (e.g., respiratory, or urinary tract infection) before the END assessment; (4) Comorbid chronic diseases such as chronic obstructive pulmonary disease, chronic heart failure, or rheumatic immune system diseases.

Written informed consent was obtained from all patients or their legal guardians before tenecteplase administration. This study was conducted in accordance with the Declaration of Helsinki. According to China’s Ethical Review Approaches for Biomedical Research Involving Humans (2016), Article 39(1) (10), informed consent was not required for this retrospective study. The study protocol was approved by the Medical Ethics Committee of Anhui No. 2 Provincial People’s Hospital (approval no. R 2025-095).

### 2.2 Imaging protocol and definition of DWI time

All patients underwent non-contrast CT (to exclude hemorrhage), routine blood tests, and coagulation function tests immediately upon admission, followed promptly by intravenous tenecteplase thrombolysis. Brain MRI-DWI (Siemens 3.0 T) was performed within 72 h after thrombolysis. The time from symptom onset to DWI completion (in hours) was recorded, and the difference between the END and non-END groups within the training set was compared.

### 2.3 Treatment protocol

According to the Chinese Stroke Association Guidelines for Clinical Management of Ischemic Cerebrovascular Diseases: Executive Summary and 2023 Update (11), intravenous thrombolysis was administered within 4.5 h of symptom onset in eligible patients without contraindications. Tenecteplase was given as an intravenous bolus at a dose of 0.25 mg/kg (maximum dose ≤25 mg). At 24 h after thrombolysis, head CT was repeated; after excluding hemorrhagic transformation, dual antiplatelet therapy with aspirin and clopidogrel was given for 2 weeks, along with intensive lipid-lowering therapy with atorvastatin.

### 2.4 Blood pressure monitoring and calculation of blood pressure variability

Blood pressure measurements were performed by trained and qualified resident physicians or specialized nurses using an automatic upper arm blood pressure monitor (HEM-4030, OMRON Life Science Co., Ltd., Kyoto, Japan). Measurements were taken with the patient in the supine position on the non-paralyzed upper arm. Blood pressure measured before tenecteplase administration was recorded as the baseline blood pressure. After thrombolysis, blood pressure was monitored every hour for 24 h. The 24-h mean blood pressure and coefficient of variation (CV = SD/mean × 100) were calculated, excluding the baseline blood pressure.

### 2.5 Collection of clinical and laboratory data

Clinical data collected included age, sex, body mass index, medical history (prior stroke/TIA, hypertension, diabetes mellitus, coronary artery disease, smoking history), culprit artery (LSA or PPA), onset-to-needle time (ONT), door-to-needle time (DNT), baseline National Institutes of Health Stroke Scale (NIHSS) score, baseline systolic blood pressure, baseline diastolic blood pressure, 24-h mean systolic blood pressure, 24-h systolic blood pressure coefficient of variation, 24-h mean diastolic blood pressure, and 24-h diastolic blood pressure coefficient of variation.

Blood samples were collected immediately after admission for measurement of neutrophil count, lymphocyte count, monocyte count, hematocrit, hemoglobin, red blood cell distribution width, platelet count, and fibrinogen. On the morning of the day after admission, fasting venous blood was collected for measurement of creatinine, uric acid, C-reactive protein, fasting blood glucose (FBG), hemoglobin A1c (HbA1c), triglycerides, low-density lipoprotein cholesterol (LDL-C), high-density lipoprotein cholesterol (HDL-C), blood urea nitrogen, and albumin.

The systemic inflammatory response index (SIRI) and blood urea nitrogen to albumin ratio (BAR) were calculated as follows: SIRI = neutrophil count × monocyte count/lymphocyte count, BAR = blood urea nitrogen (mg/dL)/albumin (g/dL).

### 2.6 Definition of END

Early neurological deterioration (END) was defined as an increase of ≥1 point in the motor items of the NIHSS or an increase of ≥2 points in the total NIHSS score within 72 h of symptom onset compared with the admission assessment (12). The determination was made jointly by two specialized neurologists who had received standardized training.

### 2.7 Statistical analysis

Data were analyzed using SPSS 26.0 and R software (version 4.5.2). Normally distributed continuous variables were expressed as mean ± standard deviation (SD), non-normally distributed continuous variables as median (interquartile range [IQR]), and categorical variables as percentages. Group comparisons were performed using the t-test, Wilcoxon rank-sum test, or chi-square test as appropriate. A two-sided *p* < 0.05 was considered statistically significant.

LASSO regression was performed using the R package “glmnet.” The optimal lambda (*λ*) was determined by 10-fold cross-validation to select the most parsimonious feature subset. Multivariate logistic regression was then conducted. A nomogram was constructed using the R package “rms.” Receiver operating characteristic (ROC) curves were generated using the “pROC” package. Calibration of the model was assessed by the Hosmer–Lemeshow test using the “Resource Selection” package. To ensure stability, internal validation was performed using 10-fold cross-validation with the “caret” package. Clinical impact curves were plotted using the “dcurves” package.

To further correct for potential overfitting due to variable selection and model training using the same dataset, we performed optimism correction with 500 bootstrap resamples. In each bootstrap iteration, the entire process of variable selection and logistic regression modeling was repeated. The difference in AUC between the bootstrap sample and the original dataset was calculated as the optimism for that iteration. The mean optimism over the 500 iterations was subtracted from the apparent AUC to obtain the corrected AUC, and the 95% confidence interval for the corrected AUC was estimated using the percentile method. Calibration curves were also optimism-corrected using the bootstrap method.

Model discrimination, calibration, and clinical utility were evaluated using ROC curves with the area under the curve (AUC), the Hosmer–Lemeshow goodness-of-fit test, calibration curves, and clinical impact curves.

External validation was performed using the fixed-coefficient method: the final logistic regression model (including the intercept and regression coefficients for the four variables) fitted in the training set was directly applied to the external validation set. The predicted probability was calculated for each patient in the validation set, and ROC and calibration curves were plotted accordingly to assess the generalizability of the model.

## 3 Results

**Figure 1:**
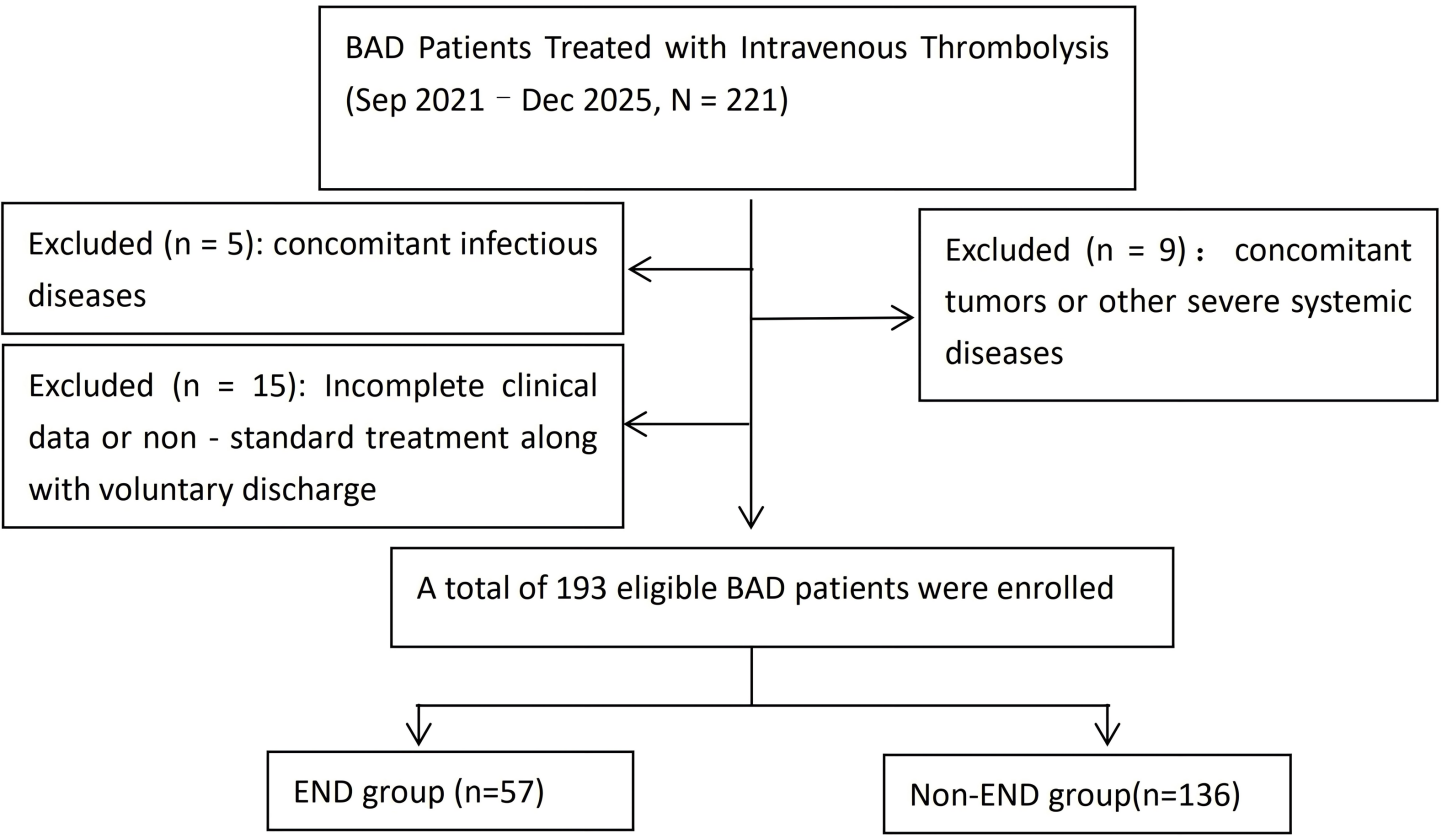
Enrollment flowchart.

A total of 229 patients with BAD receiving intravenous thrombolysis were initially enrolled. After excluding 7 patients with a history of cerebral infarction and significant residual sequelae (pre-thrombolysis mRS score ≥2), 5 patients with infectious diseases, 9 patients with tumors or other severe systemic diseases, and 15 patients with incomplete clinical data or non-standard treatment who discharged themselves against medical advice, 193 patients were finally included in the analysis. The mean age was 64.11 ± 8.57 years, and 109 were male. Among them, 57 patients developed END (END group) and 136 did not (non-END group) (Figure 1). Using the same inclusion and exclusion criteria, 265 patients were collected for external validation (83 END, 182 non-END).

### 3.1 Comparison of baseline characteristics between the training set and external validation set

There were statistically significant differences between the training set and the external validation set in age (64.11 ± 8.57 years vs. 65.75 ± 8.25 years, *p* = 0.040), onset-to-needle time (ONT: 190 min vs. 203 min, *p* < 0.001), and door-to-needle time (DNT: 58 min vs. 56 min, *p* < 0.001). No significant differences were observed in other variables, including sex, body mass index, medical history (prior stroke/TIA, hypertension, diabetes, coronary artery disease, alcohol use, smoking), culprit artery(LSA/PPA), baseline NIHSS score, admission blood pressure, 24-h blood pressure parameters, or laboratory findings (routine blood tests, blood biochemistry, inflammatory markers SIRI and BAR) (all *p* > 0.05). The overall comparability between the two sets was good, supporting the use of the external validation set (Table 1).

**Table 1:** Comparison of baseline characteristics between the training set and external validation set.

Variable	Training set (*n* = 193)	External validation set (*n* = 265)	t/χ^2^/Z	*p*
Age (years, mean ± SD)	64.11 ± 8.57	65.75 ± 8.25	2.063	0.040
Sex, *n* (%)			0.818	0.366
Male	109 (56.48%)	162 (61.13%)		
Female	84 (43.52%)	103 (38.87%)		
BMI (mean ± SD)	25.08 ± 3.80	24.87 ± 2.73	0.685	0.494
Medical history, *n* (%)				
Stroke or TIA	37 (19.17%)	53 (20.00%)	0.01	0.919
Hypertension	120 (62.18%)	160 (60.38%)	0.086	0.770
Diabetes	63 (32.64%)	83 (31.32%)	0.039	0.843
CAD	52 (26.94%)	87 (32.83%)	1.563	0.211
Alcohol Use	57 (29.53%)	83 (31.32%)	0.094	0.759
Smoking	79 (40.93%)	92 (34.72%)	1.882	0.170
Culprit artery, *n* (%)			0.335	0.563
LSA	151 (78.238%)	200 (75.47%)		
PPA	42 (21.762%)	65 (24.53%)		
ONT (min, median [IQR])	190 (179–207)	203 (185–214)	3.758	<0.001
DNT (min, median [IQR])	58 (55–61)	56 (53–59)	5.486	<0.001
NIHSS (score, median [IQR])	5 (4–6)	5 (3–6)	0.592	0.546
BP at admission				
SBP (mmHg, mean ± SD)	154.56 ± 7.17	155.31 ± 9.49	0.922	0.357
DBP (mmHg, mean ± SD)	90.71 ± 7.33	90.57 ± 7.56	0.193	0.847
Mean-24hSBP (mmHg, mean ± SD)	151.97 ± 5.92	151.83 ± 5.32	0.190	0.849
CV-24hSBP (mean ± SD)	6.09 ± 0.68	6.06 ± 0.66	0.269	0.788
Mean-24hDBP (mmHg, mean ± SD)	90.35 ± 6.73	90.06 ± 6.20	0.476	0.634
CV-24hDBP (mean ± SD)	5.46 ± 0.99	5.52 ± 1.02	0.666	0.506
Laboratory data				
HCT (median [IQR])	0.42 (0.37–0.46)	0.41 (0.37–0.46)	0.317	0.751
Hb (g/L, mean ± SD)	125.57 ± 12.31	125.02 ± 12.17	0.473	0.636
RDW (%, median [IQR])	13.83 (13.24–14.62)	13.83 (13.22–14.63)	0.123	0.902
PLT (×10^12^, median [IQR])	180 (159–207)	182 (159–207)	0.047	0.963
FIB (g/L, mean ± SD)	2.61 ± 0.33	2.63 ± 0.32	0.648	0.517
Crea (μmol/L, median [IQR])	72.00 (55.00–92.00)	73.00 (58.00–93.00)	0.676	0.499
UA (μmol/L median [IQR])	412.00 (346.00–456.00)	412.00 (345.00–465.00)	0.114	0.909
CRP (mg/L, median [IQR])	13.65 (10.79–17.46)	13.53 (10.99–16.95)	0.358	0.720
FBG (mmol/L, median [IQR])	6.60 (5.60–7.50)	6.70 (5.90–7.60)	0.856	0.392
HbA1c (% median [IQR])	6.53 (5.85–7.24)	6.55 (5.95–7.23)	0.913	0.361
TG (mmol/L, median [IQR])	2.96 (2.36–3.71)	2.99 (2.45–3.72)	0.700	0.484
LDL-C (mmol/L, mean ± SD)	2.80 ± 0.72	2.93 ± 0.67	1.894	0.059
HDL-C (mmol/L, mean ± SD)	1.47 ± 0.44	1.49 ± 0.44	0.613	0.54
SIRI (median [IQR])	1.40 (1.13–1.78)	1.49 (1.16–1.76)	0.411	0.682
BAR (median [IQR])	6.08 (4.68–7.07)	6.13 (5.17–6.98)	0.686	0.493

END, early neurological deterioration; BMI, body mass index; CAD, coronary artery disease; LSA, lenticulostriate artery; PPA, paramedian pontine artery; ONT, onset-to-needle time; DNT, door-to-needle time; NIHSS, National Institutes of Health Stroke Scale; SBP, systolic blood pressure; DBP, diastolic blood pressure; HCT, hematocrit; Hb, hemoglobin; RDW, red blood cell distribution width; PLT, platelet count; FIB, fibrinogen; Crea, creatinine; UA, uric acid; CRP, C-reactive protein; FBG, fasting blood glucose; HbA1c, glycated hemoglobin; TG, triglycerides; LDL-C, low-density lipoprotein cholesterol; HDL-C, high-density lipoprotein cholesterol; SIRI, systemic inflammatory response index; BAR, blood urea nitrogen to albumin ratio.

### 3.2 Comparison of demographic, clinical, and laboratory data between the END and non-END groups in the training set

No significant differences were found between the two groups in age, sex, body mass index, history of stroke/TIA, hypertension, diabetes, coronary artery disease, alcohol use, smoking, onset-to-DWI time, culprit artery (LSA/PPA), ONT, DNT, baseline NIHSS score, baseline systolic and diastolic blood pressure, 24-h mean diastolic blood pressure, 24-h diastolic blood pressure coefficient of variation, hematocrit, hemoglobin, red blood cell distribution width, platelet count, fibrinogen, creatinine, uric acid, C-reactive protein, glycated hemoglobin, triglycerides, low-density lipoprotein cholesterol, or high-density lipoprotein cholesterol (all *p* > 0.05). The END group had significantly higher 24-h mean systolic blood pressure, 24-h systolic blood pressure coefficient of variation (CV-24hSBP), fasting blood glucose (FBG), SIRI, and BAR than the non-END group (all *p* < 0.05) (Table 2).

**Table 2:** Comparison of clinical and laboratory data between the END group and non-END group in the training set.

Variable	Total (*n* = 193)	Non-END group (*n* = 136)	END group (*n* = 57)	t/χ^2^/Z value	*p*
Age (years, mean ± SD)	64.11 ± 8.57	64.10 ± 8.92	64.1 ± 7.76	0.028	0.978
Sex, *n* (%)				0.540	0.463
Male	109 (56.48%)	74 (54.41%)	35 (61.40%)		
Female	84 (43.52%)	62 (45.59%)	22 (38.60%)		
BMI (mean ± SD)	25.08 ± 3.80	24.92 ± 3.79	25.45 ± 3.83	0.872	0.385
Medical history, *n* (%)					
Stroke or TIA	37 (19.17%)	26 (19.12%)	11 (19.30%)	0.000	1.000
Hypertension	120 (62.18%)	82 (60.29%)	38 (66.67%)	0.449	0.503
Diabetes	63 (32.64%)	43 (31.62%)	20 (35.09%)	0.090	0.764
CAD	52 (26.94%)	35 (25.74%)	17 (29.83%)	0.165	0.685
Alcohol Use	57 (29.53%)	39 (28.68%)	18 (31.58%)	0.053	0.818
Smoking	79 (40.93%)	55 (40.44%)	24 (42.11%)	0.003	0.957
Onset-to-DWI time (h, median [IQR])	44 (35.00–52.00)	47.00 (37.00–52.25)	38.00 (30.00–52.00)	1.847	0.065
Culprit artery, *n* (%)				0.001	0.971
LSA	151 (78.238%)	107 (78.676%)	44 (77.193%)		
PPA	42 (21.762%)	29 (21.324%)	13 (22.807%)		
ONT (min, median [IQR])	190 (179–207)	190 (180–209)	192 (179–203)	0.294	0.769
DNT (min, median [IQR])	58 (55–61)	58 (55.75–61)	58 (55–61)	0.184	0.854
NIHSS (score, median [IQR])	5 (4–6)	5 (3.75–6)	5 (4–6)	0.284	0.772
BP at admission					
SBP (mmHg, mean ± SD)	154.56 ± 7.17	154.125 ± 7.254	155.59 ± 6.93	1.303	0.194
DBP (mmHg, mean ± SD)	90.71 ± 7.33	90.61 ± 7.33	90.95 ± 7.40	0.291	0.771
Mean-24hSBP (mmHg, mean ± SD)	151.97 ± 5.92	151.39 ± 5.69	153.34 ± 6.27	2.108	0.036
CV-24hSBP (mean ± SD)	6.09 ± 0.68	5.95 ± 0.69	6.26 ± 0.61	2.936	0.004
Mean-24hDBP (mmHg, mean ± SD)	90.35 ± 6.73	90.47 ± 6.25	90.06 ± 7.80	0.388	0.698
CV-24hDBP (mean ± SD)	5.46 ± 0.99	5.42 ± 1.03	5.53 ± 0.91	0.702	0.483
Laboratory data					
HCT (median [IQR])	0.42 (0.37–0.46)	0.41 (0.36–0.46)	0.43 (0.39–0.48)	1.376	0.168
Hb (g/L, mean ± SD)	125.57 ± 12.31	125.11 ± 12.72	126.67 ± 11.31	0.800	0.424
RDW (%, median [IQR])	13.83 (13.24–14.62)	13.80 (13.12–14.59)	13.96 (13.44–14.70)	1.788	0.074
PLT (×10^12^, median [IQR])	180.00(159.00–207.00)	177.00 (157.75–206.00)	186.00 (163.00–210.00)	1.099	0.272
FIB (g/L, mean ± SD)	2.61 ± 0.33	2.635 ± 0.33	2.55 ± 0.33	1.551	0.123
Crea (μmol/L, median [IQR])	72.00 (55.00–92.00)	72.00 (53.75–92.00)	72.00 (58.00–87.00)	0.523	0.601
UA (μmol/L median [IQR])	412.00 (346.00–456.00)	406.50 (345.00–447.75)	421.00 (356.00–473.00)	1.230	0.218
CRP (mg/L, median [IQR])	13.65 (10.79–17.46)	13.63 (10.94–16.62)	13.65 (10.54–18.56)	0.893	0.372
FBG (mmol/L, median [IQR])	6.60 (5.60–7.50)	6.50 (5.58–7.40)	7.20 (6.30–8.30)	2.090	0.036
HbA1c (% median [IQR])	6.53 (5.85–7.24)	6.53 (5.86–7.18)	6.60 (5.80–7.37)	0.578	0.563
TG (mmol/L, median [IQR])	2.96 (2.36–3.71)	2.98 (2.36–3.72)	2.88 (2.36–3.53)	0.606	0.545
LDL-C (mmol/L, mean ± SD)	2.80 ± 0.72	2.75 ± 0.71	2.93 ± 0.75	1.619	0.107
HDL-C (mmol/L, mean ± SD)	1.47 ± 0.44	1.48 ± 0.43	1.45 ± 0.44	0.480	0.632
SIRI (median [IQR])	1.40 (1.13–1.78)	1.30 (1.02–1.71)	1.69 (1.34–1.98)	4.394	<0.001
BAR (median [IQR])	6.08 (4.68–7.07)	5.79 (4.50–6.76)	6.52 (5.45–7.79)	3.178	0.001

END, Early Neurological Deterioration; BMI, Body Mass Index; CAD, Coronary Artery Disease; LSA, lenticulostriate artery; PPA, Pontine Paramedian Arteries; ONT, Onset-to-Needle Time; DNT, Door-to-Needle Time; NIHSS, National Institutes of Health Stroke Scale; SBP, systolic pressure; DBP, diastolic pressure; CV-24hSBP, 24-h systolic blood pressure coefficient of variation; CV-24hDBP, 24-h diastolic blood pressure coefficient of variation; Mean-24hSBP, 24-h mean systolic blood pressure; Mean-24hDBP, 24-h mean diastolic blood pressure. HCT, hematocrit; Hb, hemoglobin; RDW, Red Blood Cell Distribution Width; PLT, platelet; FIB, fibrinogen; Crea, creatinine; UA, uric acid; CRP, C-reactive protein; FBG, fasting blood glucose; HbA1c, Hemoglobin A1c; TG, Triglyceride; LDL-C, Low-Density Lipoprotein Cholesterol; HDL-C, High-Density Lipoprotein Cholesterol; SIRI, Systemic Inflammatory Response Index; BAR, blood urea nitrogen to albumin ratio.

### 3.3 Variable selection using LASSO regression

**Figure 2:**
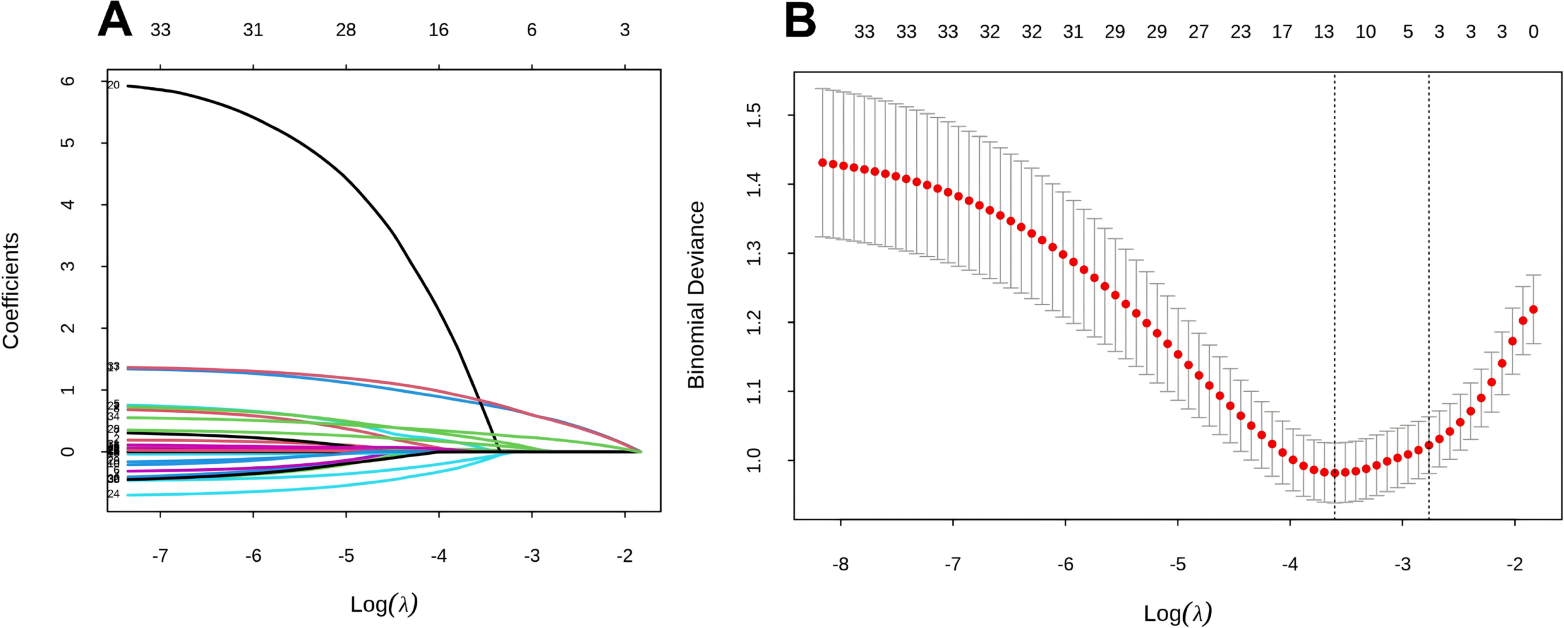
Variable selection via LASSO regression: **(A)** The coefficient distribution plot of candidate predictive variables plotted against the logarithm (*λ*) series. **(B)** Selection of the optimal penalty coefficient in the LASSO regression.

LASSO regression was performed with END as the dependent variable to reduce dimensionality and select the most representative predictors of END. The coefficient path plot showed that as the penalty parameter *λ* changed, the coefficients of the independent variables were gradually shrunk to zero (Figure 2A). The cross-validation curve (Figure 2B) plotted the log(λ) against the mean squared error (MSE). The left dashed line corresponds to the λ value with the minimum MSE, and the right dashed line corresponds to the λ value with the minimum MSE plus one standard error (λ.1se), which was chosen as the optimal penalty coefficient. Four variables with non-zero coefficients were selected as the optimal feature subset: CV-24hSBP, FBG, SIRI, and BAR (Figures 2A,B).

### 3.4 Logistic regression analysis of factors associated with END and nomogram construction

The four variables selected by LASSO regression were entered into a multivariate logistic regression analysis. The results showed that CV-24hSBP (OR = 2.704, 95% CI: 1.471–4.967, *p* = 0.001), FBG (OR = 1.253, 95% CI: 1.016–1.547, *p* = 0.034), SIRI (OR = 3.140, 95% CI: 1.584–6.223, *p* < 0.001), and BAR (OR = 1.480, 95% CI: 1.207–1.815, *p* < 0.001) were all independent factors associated with END. Among these, SIRI had the highest risk (OR>3), followed by CV-24hSBP (OR > 2), while FBG and BAR showed relatively lower risks (OR < 2). All variables were statistically significant (*p* < 0.05), indicating their important clinical value in predicting END (Table 3). Based on the multivariate logistic regression analysis, the predictive model equation was constructed as follows: Logit(p) = −12.884 + 0.995 × (CV-24hSBP) + 0.226 × (FBG) + 1.144 × (SIRI) + 0.392 × (BAR), where p denotes the predicted probability of END. The linear predictor (LP) for each patient was calculated using this equation, and the predicted probability of END was then obtained as *p* = 1/(1 + e^{−LP}).

**Table 3:** Univariate and multivariate logistic regression analysis of END in patients with BAD after tenecteplase intravenous thrombolysis.

Univariate logistic regression					Multivariate logistic regression			
Variable	OR	95% CI (lower)	95% CI (upper)	*p*	OR	95% CI (lower)	95% CI (upper)	*p*
BAR	1.545	1.272	1.877	<0.001	1.480	1.207	1.815	<0.001
FBG	1.280	1.061	1.543	0.009	1.253	1.016	1.547	0.034
SIRI	3.665	1.993	6.737	<0.001	3.140	1.584	6.223	<0.001
CV-24hSBP	3.542	2.053	6.112	<0.001	2.704	1.471	4.967	0.001

BAR, blood urea nitrogen to albumin ratio; FBG, fasting blood glucose; SIRI, systemic inflammatory response index; CV-24hSBP, coefficient of variation of 24-h systolic blood pressure; OR, odds ratio; CI, confidence interval.

**Figure 3:**
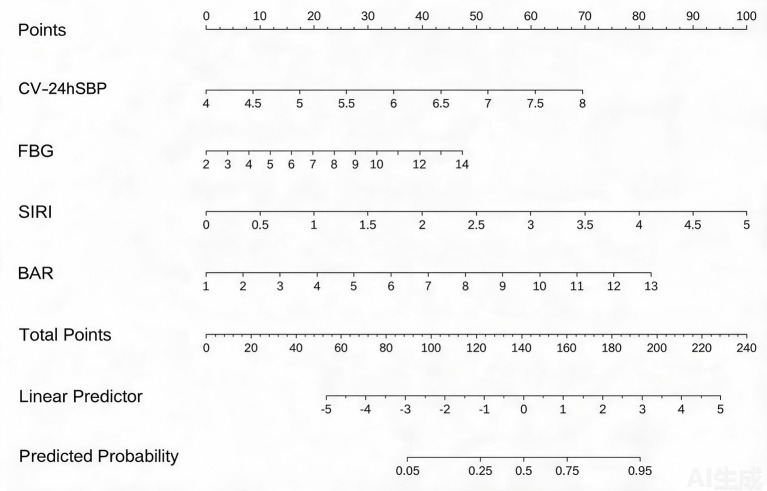
Nomogram predicting END in BAD patients after intravenous thrombolysis with tenecteplase. END, early neurological deterioration; BAD, branch atheromatous disease; CV-24hSBP, coefficient of variation of 24-h systolic blood pressure; FBG, fasting blood glucose; SIRI, systemic inflammatory response index; BAR, blood urea nitrogen to albumin ratio; Points, assigned score for each predictor; Total Points, sum of all points; Risk, predicted probability of END.

A nomogram was constructed based on these four predictors. Each predictor was assigned a point value according to its contribution. The total points, summed across all predictors, corresponded to a risk axis value representing the probability of END. Higher total points indicated a higher risk of END after tenecteplase thrombolysis in BAD patients (Figure 3).

### 3.5 Evaluation of model discrimination, calibration, and clinical utility

**Figure 4:**
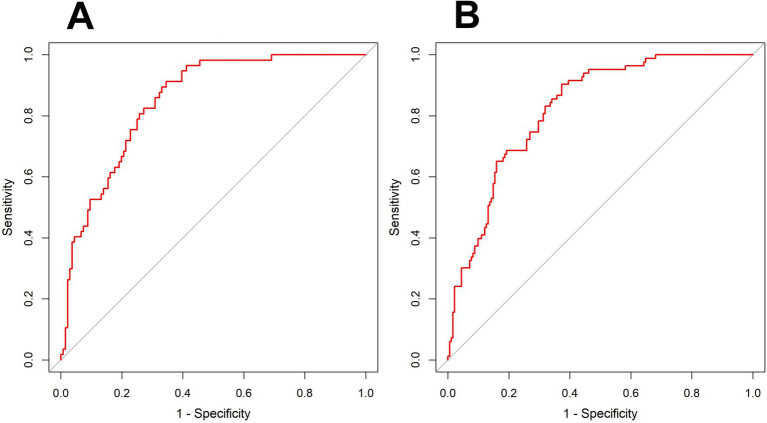
ROC curves of the nomogram for predicting END. **(A)** In the training set, the ROC curve of the combined four predictors yielded an AUC of 0.850 (95% CI: 0.796–0.904). After 500 bootstrap optimism corrections, the corrected AUC was 0.837 (95% CI: 0.780–0.889). **(B)** In the external validation set, the ROC curve of the fixed-coefficient model derived from the training set yielded an AUC of 0.824 (95% CI: 0.778–0.879).

In the training set, the ROC curve of the model showed an apparent area under the curve (AUC) of 0.850 (95% CI: 0.796–0.904), with a Youden index of 0.567, corresponding to a sensitivity of 0.912 and a specificity of 0.654 (Figure 4). To correct for overfitting, optimism correction was performed using 500 bootstrap resamples. The mean optimism was 0.013, and the corrected AUC was 0.837 (95% CI: 0.780–0.889), indicating good discriminative ability with minimal overfitting. In the external validation set, the apparent AUC was 0.824 (95% CI: 0.778–0.879), suggesting good generalizability of the model (Figures 4A,B).

**Figure 5:**
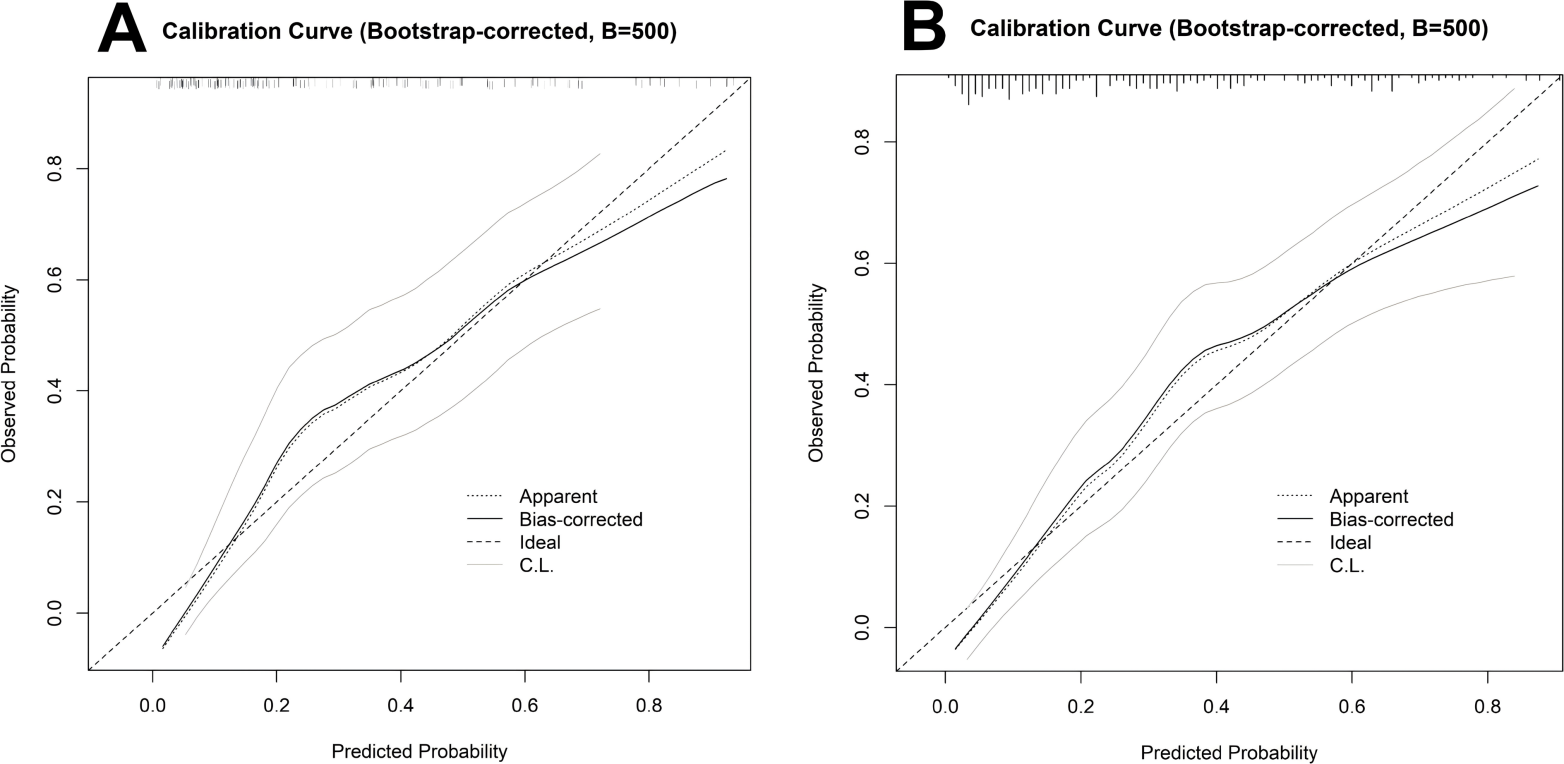
Calibration curves of the nomogram for predicting END in BAD patients after tenecteplase thrombolysis in **(A)** Training set and **(B)** External validation set. END, early neurological deterioration; BAD, branch atheromatous disease; ideal, 45°diagonal line representing perfect calibration; apparent, unadjusted calibration curve; bias-corrected, calibration curve after 500 bootstrap optimism corrections; C.L., 95% confidence limits of the bias-corrected curve.

The Hosmer–Lemeshow test in the training set yielded a *p* value of 0.581, indicating good model fit. The calibration curve showed high agreement between predicted probabilities and observed risks: the apparent calibration curve had an intercept of 0.040, a slope of 0.912, and *R*^2^ = 0.967. After 500 bootstrap optimism corrections, the bias-corrected curve was very close to the ideal line, with an intercept of 0.060 (95% CI: 0.031–0.090), a slope of 0.853 (95% CI: 0.799–0.907), and *R*^2^ = 0.955, indicating that the model maintained good predictive accuracy after calibration with only slight overfitting. In the external validation set, the apparent calibration curve had an intercept of 0.0308 (95% CI: 0.0077–0.0538), a slope of 0.9162 (95% CI: 0.8711–0.9612), and *R*^2^ = 0.9721 (Figures 5A,B).

**Figure 6:**
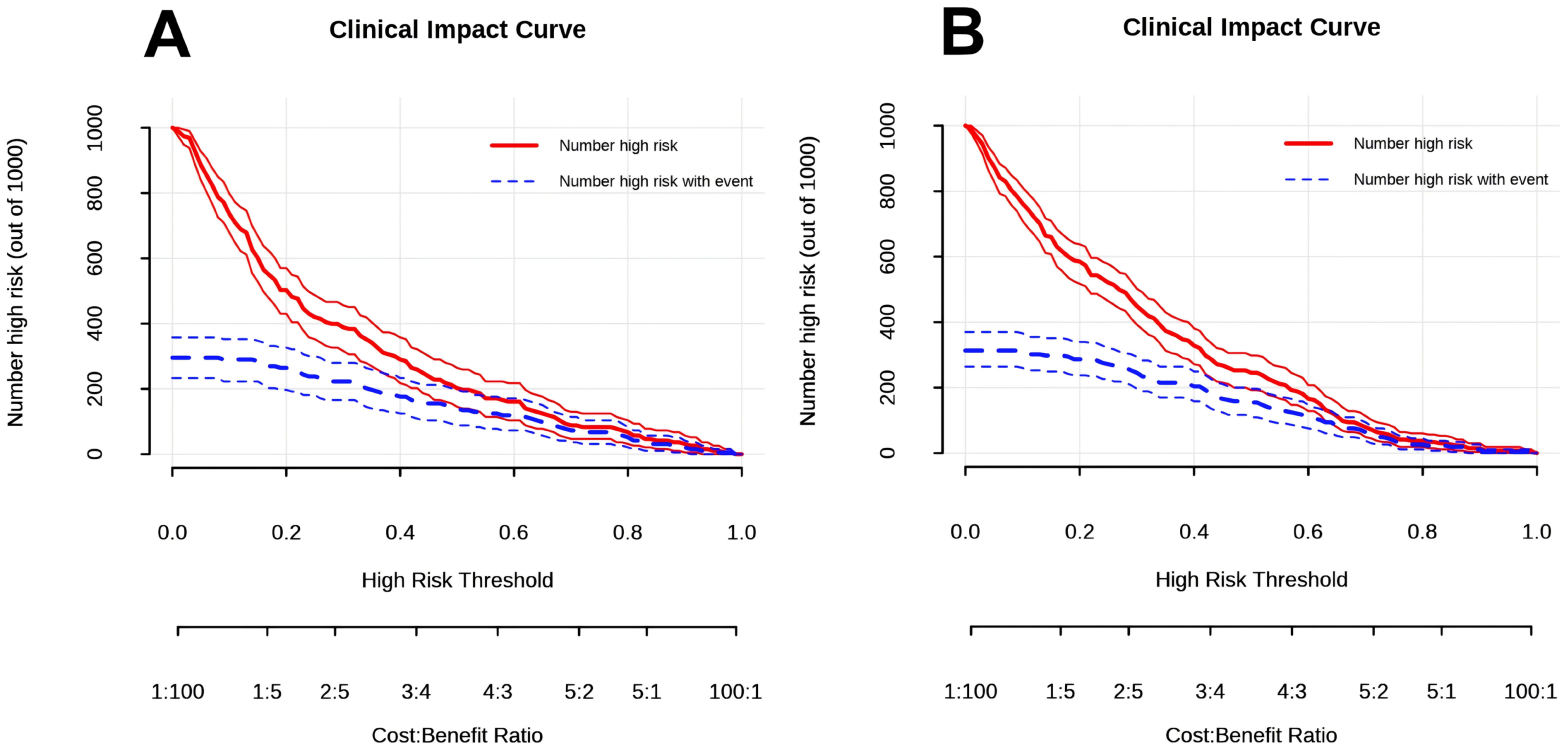
Clinical impact curves (CIC). The red line shows model-predicted high-risk patients, and the blue line shows observed END cases. **(A)** Training set. **(B)** External validation set.

Clinical impact curves (CIC) were used to evaluate the clinical utility of the predictive model. The x-axis represents the high-risk threshold, and the y-axis represents the number of END cases per 1,000 individuals. The red line indicates the number of patients predicted to be at high risk by the model, and the blue line indicates the actual number of END cases. The curves showed that at low threshold values, the number of predicted high-risk patients was relatively large; as the threshold increased, the number of predicted high-risk patients gradually decreased. At a threshold of 0.5, the predicted high-risk number dropped to 200, and the number of true positives was approximately 160, with predicted values close to actual values, indicating that the model has good predictive performance and clinical utility (Figures 6A,B).

## 4 Discussion

The causes of END in BAD patients are multifactorial and cannot be adequately explained or predicted by a single factor. Considering multiple factors often improves predictive accuracy. We used LASSO regression to efficiently select the most predictive variables from a large set of potential risk factors, thereby enhancing the precision and predictive power of the model and increasing its practical value for clinical decision-making. This study identified 24-h systolic blood pressure variability, fasting blood glucose, SIRI, and BAR as predictors of early neurological deterioration (END) in patients with branch atheromatous disease (BAD) after intravenous tenecteplase thrombolysis. Receiver operating characteristic (ROC) curve analysis showed that our predictive model had good performance (AUC = 0.850) and good generalizability (external validation set AUC = 0.824). It should be emphasized that this model relies on blood pressure variability measured over 24 h after thrombolysis; therefore, it is not suitable for pre-thrombolysis or ultra-early prediction but can serve as an in-hospital early risk stratification tool. Based on the ROC analysis, the optimal cut-off value was determined to be 0.168 (maximizing the Youden index). For ease of clinical application, we suggest using a predicted probability >0.2 as a high-risk trigger threshold and a probability <0.1 as a low-risk threshold. The clinical impact curves also supported good predictive calibration of the model around this threshold. Clinicians can use the predicted probability of END to guide management: for high-risk patients, more attention should be given, including intensified neurological monitoring, shorter intervals for repeat imaging, or adoption of more aggressive blood pressure management; for low-risk patients, monitoring intensity may be appropriately reduced to optimize resource utilization.

Our study showed that CV-24hSBP after tenecteplase thrombolysis in BAD patients was significantly associated with END. A recent study also demonstrated that increased systolic blood pressure variability within 72 h of onset is independently associated with an increased risk of poor clinical outcomes in BAD patients (13). Although blood pressure variability is lower in BAD patients than in those with large-artery atherosclerotic ischemic stroke (14), increased systolic blood pressure variability still adversely affects the prognosis of BAD patients (15). Previous studies have found that some BAD patients exhibit, on DWI, one or more additional lesions in the same perforating artery territory besides the main lesion, a feature termed the “island sign.” The island sign is particularly closely associated with END in patients with LSA involvement (16). These findings suggest that hemodynamics play an important role in the development and progression of END in BAD patients, and that excessive fluctuation in systolic blood pressure should be controlled in the early stages of symptom onset.

Our study identified fasting blood glucose as a factor associated with END after tenecteplase thrombolysis in BAD patients. Approximately 50% of patients with acute ischemic stroke develop stress hyperglycemia. Elevated blood glucose levels within 24 h of admission are associated with END in acute ischemic stroke patients receiving intravenous thrombolysis (17). Even in non-diabetic patients with acute cerebral infarction, normal fasting blood glucose on the morning after thrombolysis is a protective factor for long-term prognosis (18). Elevated baseline fasting blood glucose is an independent risk factor for 1-year all-cause mortality in nondiabetic patients with acute cerebral infarction (19).

Our study found a significant positive correlation between SIRI and END after tenecteplase thrombolysis in BAD patients. Logistic regression analysis indicated that SIRI was an independent factor associated with END after thrombolysis, and ROC curve analysis showed that SIRI had high predictive value for END. Many inflammatory markers, such as the serum uric acid-to-creatinine ratio (20), BAR (21), neutrophil-to-lymphocyte ratio (22), and RDW (23), have prognostic value in stroke patients. Previous studies have shown that SIRI plays an important role in the prognostic assessment of cardiovascular diseases, tumors, and infectious diseases (24–26). Recent studies have found that SIRI is associated with all-cause mortality in stroke patients, with higher SIRI corresponding to increased mortality. SIRI is a promising low-grade inflammatory marker for predicting stroke prognosis, and its predictive ability is superior to that of neutrophil-to-lymphocyte ratio, platelet-to-lymphocyte ratio, lymphocyte-to-monocyte ratio, and RDW (27). In patients with mild acute ischemic stroke receiving intravenous thrombolysis, elevated SIRI is significantly associated with poor 3-month outcomes, and a higher SIRI value can be used to predict poor clinical outcomes in mild acute ischemic stroke patients after intravenous thrombolysis (28).

Our study found that BAR was significantly positively correlated with END after tenecteplase thrombolysis in BAD patients. BAR is a comprehensive parameter reflecting renal function, inflammatory status, nutritional status, and endothelial function. Studies have shown that BAR is closely associated with various diseases, including various cancers, pneumonia, sepsis, and pulmonary and cardiovascular diseases. Recent research further revealed that BAR is significantly associated with cerebral small-vessel disease in health check-up participants and is a key indicator for identifying individuals at high risk of cerebral small-vessel disease (29). In patients with acute ischemic stroke, combined deterioration of nutritional and renal function is closely associated with a significantly increased risk of all-cause mortality, and BAR can serve as an effective predictor of all-cause mortality in acute ischemic stroke patients (21). Based on data from the third China National Stroke Registry (August 2015 to March 2018), BAR was confirmed to be a factor associated with 1-year all-cause mortality in acute ischemic stroke patients, with higher levels closely associated with increased mortality risk (30). Data from South Korea (31) showed a complex, non-linear relationship between BAR and 3-month poor outcomes in acute ischemic stroke patients, with an inflection point at 2.86. To the left of this inflection point, the association was not statistically significant; to the right, the risk of poor outcomes increased significantly by 9.47%.

Previous prediction models for END after intravenous thrombolysis have been mostly based on general acute ischemic stroke populations and have primarily included conventional clinical variables such as age and baseline NIHSS score (12, 32), lacking specificity for BAD. The incremental value of our model lies in the following two aspects. First, it incorporates hemodynamic indicators. Previous studies have focused mainly on pre-thrombolysis single blood pressure measurements (33), whereas our study identified CV-24hSBP as an independent predictor in BAD patients, suggesting that blood pressure variability is more pathophysiologically relevant than a single systolic blood pressure value. Second, it combines inflammatory-nutritional composite markers. While previous models have mostly used single inflammatory markers such as NLR (34), SIRI—as a composite inflammatory index—has been shown to have superior predictive performance over single indicators (35). Our study further included BAR, which integrates multidimensional information covering renal function, inflammatory status, nutritional status, and endothelial function, thereby providing a more comprehensive and objective risk assessment.

However, this study used only a single baseline measurement of SIRI and BAR. Although this approach is more convenient in clinical practice, the dynamic evolution of the inflammatory response suggests that a single measurement may not fully capture its changing trajectory. Future studies could explore serial measurements of SIRI and BAR at multiple time points after thrombolysis (e.g., 24 h, 48 h, and 72 h) to analyze the association between their dynamic trends and END, with the aim of constructing a predictive model with enhanced real-time risk adjustment capability.

## 5 Conclusion

Based on four independent predictors (CV-24hSBP, FBG, SIRI, and BAR), this study successfully constructed and validated a nomogram model for predicting early neurological deterioration (END) after intravenous tenecteplase thrombolysis in patients with branch atheromatous disease (BAD). The model demonstrated good discrimination (apparent AUC = 0.850, optimism-corrected AUC = 0.837) and calibration (Hosmer–Lemeshow test *p* = 0.581) in the training set, and was preliminarily validated in an external validation set (AUC = 0.824). Because CV-24hSBP requires continuous blood pressure monitoring for 24 h after thrombolysis, this model is not suitable for pre-thrombolysis or ultra-early prediction but can serve as an in-hospital early risk stratification tool. Future prospective, multicenter studies are needed to further validate the model’s generalizability and to explore the use of blood pressure variability measured over shorter monitoring windows (e.g., 6 or 12 h) to enable earlier risk prediction.

### 5.1 Limitations

First, the timing of DWI examination was not strictly standardized (24–72 h after thrombolysis). Although there was no significant difference in the time from onset to DWI between the END and non-END groups (*p* = 0.065), different time windows may still affect the interpretation of lesions.

Second, although optimism correction using 500 bootstrap resamples was performed (corrected AUC = 0.837) and an external validation set was used (AUC = 0.824), the sample size was relatively small, and all data were retrospective. Moreover, the data collection periods for the training and validation sets overlapped (both from September 2021 to October 2025), so the model’s ability to predict outcomes in future patient cohorts was not assessed. Therefore, the risk of overfitting remains, and the predictive performance may be overly optimistic. Future studies with cross-regional, international, and multicenter prospective designs are needed to further validate the applicability of this model across different populations and healthcare settings. Third, long-term functional outcomes were not evaluated. This study focused only on early neurological deterioration (END) and did not analyze functional prognosis at 3 or 6 months (e.g., mRS score). Hence, the predictive value of this model for long-term outcomes remains unclear.

Fourth, due to the retrospective design, we did not collect potential confounders such as infarct size, the “island sign” of perforating arteries, parent artery stenosis, baseline perfusion status, post-thrombolysis blood pressure management details, or some in-hospital complications. Residual confounding cannot be completely excluded, and causal inference should be made with caution.

## Data Availability

The raw data supporting the conclusions of this article will be made available by the authors, without undue reservation.
